# Highly efficient degradation of cypermethrin by a co-culture of *Rhodococcus* sp. JQ-L and *Comamonas* sp. A-3

**DOI:** 10.3389/fmicb.2022.1003820

**Published:** 2022-09-16

**Authors:** Jian He, Kaiyun Zhang, Lin Wang, Yingchun Du, Ying Yang, Cansheng Yuan

**Affiliations:** ^1^College of Rural Revitalization, Jiangsu Open University, Nanjing, China; ^2^Key Laboratory of Agricultural Environmental Microbiology, Ministry of Agriculture, College of Life Sciences, Nanjing Agricultural University, Nanjing, China

**Keywords:** cypermethrin-degrading, *Rhodococcus* sp., *Comamonas* sp., catabolic pathway, 3-Phenoxybenzoic acid

## Abstract

Cypermethrin is an important synthetic pyrethroid pesticide that widely used to control pests in agriculture. However, extensive use has caused its residue and the metabolite 3-phenoxybenzoic acid (3-PBA) to seriously pollute the environments and agricultural products. In this study, a highly efficient cypermethrin-degrading bacterial consortium was acclimated from long-term pyrethroid-contaminated soil. Two strains, designated JQ-L and A-3, were screened from the consortium, and identified as *Rhodococcus* sp. and *Comamonas* sp., respectively. Strain JQ-L transformed 100 mg/L of cypermethrin to 3-PBA within 60 h of incubation; however, 3-PBA could not be further degraded by the strain. Strain A-3 utilized 3-PBA as sole carbon for growth, and completely degraded 100 mg/L of 3-PBA within 15 h of incubation. Co-culture of JQ-L and A-3 completely degraded 100 mg/L of cypermethrin within 24 h of incubation. Furthermore, a complete catabolic pathway of cypermethrin and the metabolite 3-PBA by the co-culture was proposed. This study provided a promising strategy for efficient elimination of cypermethrin residue-contaminated environments and agricultural products.

## Introduction

Cypermethrin [cyano-(3-phenoxyphenyl)methyl (1R,3S)-3-(2,2-dichloroethenyl)-2,2-dimethylcyclopropane-1-carboxylate] is an important synthetic pyrethroid pesticide (Tallur et al., 2008; Lin et al., 2011; Chen et al., 2012a; Chen et al., 2014; Zhan et al., 2020). Due to its broad spectrum and high efficiency, cypermethrin was widely used to control various pests in cotton, rice, corn, soybean, fruit trees and vegetables (Dorman and Beasley, 1991; Katsuda, 1999). In recent years, the usage of cypermethrin has rapidly increased and become a dominant pesticide worldwide with the restrictions of highly toxic organophosphorus and organochlorine pesticides. However, extensive usage of cypermethrin has resulted in frequent detection of its residues in the environments and agricultural products, which accumulated in human or mammal bodies through food chain (Schettgen et al., 2002; Kuivila et al., 2012; Łozowicka et al., 2012; McCoy et al., 2012). Although cypermethrin generally has lower toxicity to human or mammalian than organophosphorus and organochlorine pesticides, prolonged exposure to high concentrations of cypermethrin might cause endocrine disruption, spleen damage, and carcinogenesis (Wolansky and Harrill, 2008; Dewailly et al., 2014; Sun et al., 2014; Ye et al., 2017; Zepeda-Arce et al., 2017; Chrustek et al., 2018; Navarrete-Meneses and Pérez-Vera, 2019; Gammon et al., 2019). Furthermore, cypermethrin residue in the environments had strong acute toxicity to some non-target organisms, such as aquatic invertebrate, fish and bee (Stratton and Corke, 1982; Smith and Stratton, 1986; Jin et al., 2011). Therefore, it is urgent to develop effective technology to eliminate cypermethrin residue from the environments and agricultural products.

Biodegradation is an ideal technology for removal of cypermethrin residue because of its high efficiency, environmental friendliness and low cost (Guo et al., 2009; Lin et al., 2011; Chen et al., 2012a; Ruan et al., 2013). To date, a lot of microorganisms that were able to degrade cypermethrin have been isolated, such as *Bacillus licheniformis* B-1 (Liu et al., 2014; Zhao et al., 2016), *Bacillus cereus* ZH-3 (Chen et al., 2012b), *Sphingobium wenxiniae* JZ-1 (Guo et al., 2009; Wang et al., 2009), *Trichoderma viride* and *Aspergillus niger* (Saikia and Gopal, 2004; Zhao et al., 2016; Zhan et al., 2020). However, most of these microorganisms possessed a relatively low cypermethrin-degrading efficiency and could not completely mineralized cypermethrin due to their lack of a complete catabolic enzymes system. E.g., *Bacillus licheniformis* B-1, *Bacillus cereus* ZH-3 and *Acinetobacter junii* LH-1-1 transformed cypermethrin to 3-phenoxybenzoic acid (3-PBA), but could not further degrade 3-PBA (Chen et al., 2012b; Liu et al., 2014). 3-PBA is an endocrine disruptor due to its antiestrogenic activity and is persistent in environment; furthermore, 3-PBA is hydrophilic and therefore easier to migrate in the environment than cypermethrin (Halden et al., 1999; Han et al., 2008; Chen et al., 2011; Chen et al., 2012b; Sun et al., 2014). Therefore, simultaneous elimination of cypermethrin and the metabolite 3-PBA are crucial to environmental and human health. Co-culture of strains capable of degrading cypermethrin and 3-PBA, respectively, is an effective method to completely eliminate cypermethrin residue (Huang et al., 2018; Zhan et al., 2020). In previous reports, Liu et al found that co-culture of cypermethrin-degrading strain *Bacillus licheniformis* B-1 and 3-PBA-degrading strain *Sphingomonas* sp. SC-1 or *Aspergillus oryzae* M-4 significantly improved the degradation efficiency of cypermethrin when compared with culture of *Bacillus licheniformis* B-1 alone (Liu et al., 2014; Zhao et al., 2016; Zhao et al., 2019), and Chen et al. (2012b) found that co-culture of cypermethrin-degrading strain *Bacillus cereus* ZH-3 and 3-PBA-degrading strain *Streptomyces aureus* HP-S-01 completely mineralized cypermethrin within 72 h of incubation.

In this study, we screened two strains JQ-L and A-3 from a highly efficient cypermethrin-degrading bacterial consortium. Strain JQ-L was identified as *Rhodococcus* sp. and could transform cypermethrin to 3-PBA, while strain A-3 was identified as *Comamonas* sp. and could completely degrade 3-PBA. Co-culture of JQ-L and A-3 efficiently and completely degraded cypermethrin within 24 h of incubation. Furthermore, a complete catabolic pathway of cypermethrin by co-culture of JQ-L and A-3 was also proposed in this study.

## Materials and methods

### Chemicals and media

Cypermethrin (97%) was purchased from Jiangsu Yangnong Chemical Group Co., Ltd, 3-PBA (98%) and chromatographic grade acetonitrile were purchased from Sigma-Aldrich Chemical Co. (Shanghai, China). All other chemicals and solvents used in this study were analytical grade. Cypermethrin was dissolved in methanol as stock solutions (24 mM), and sterilized by membrane filtration (0.22 μm). The R2A medium consisted of 0.25 g/L tryptone, 0.5 g/L yeast extract and 0.5 g/L casein acid hydrolyzate. The Luria-Bertani (LB) medium consisted of 10 g/L tryptone, 5 g/L yeast extract, and 10 g/L NaCl, pH was adjusted to 7.0. Mineral salt medium (MSM) consisted of 1.0 g/L NH_4_Cl, 1.0 g/L NaCl, 1.5 g/L K_2_HPO_4_, 0.5 g/L KH_2_PO_4_ and 0.2 g/L MgSO_4_⋅7H_2_O, pH 7.0. For solid medium, 1.5% of agar was added. All medium was autoclaved at 121°C for 30 min.

### Enrichment, isolation, and identification of cypermethrin-degrading strain

The soil sample was collected from the long-term pyrethroid-contaminated site near Jiangsu Yangnong Chemical Group Co., Ltd (119° 15′ E, 32° 26′ N) in September 2020. Soil sample (10 g) was added to a 250 mL Erlenmeyer flask containing 90 mL of MSM supplemented with 0.24 mM of cypermethrin. The flask was then incubated at 30°C and 180 rpm on a rotary shaker. At certain intervals, the degradation of cypermethrin was detected using ultraviolet-visible spectrophotometer (UV/VIS) or Gas chromatography (GC) as described below. When approximately 60–70% of the added cypermethrin was degraded, 10 mL of the enrichment culture was transferred into 90 mL fresh medium. The transfer was repeated for 10 times until the enriched consortium acquired highly efficient cypermethrin-degrading ability. Then, the last round enrichment culture was serially diluted and spread on R2A plates. After incubated at 30°C for 4–6 days, colonies with different morphologies were selected and purified by the streak plate method. The abilities of the isolates to degrade cypermethrin or 3-PBA were detected by GC or high-performance liquid chromatography (HPLC), respectively, as described below.

The acquired isolates were characterized and identified by morphological, physiological and biochemical characteristics as well as 16S rRNA gene sequence analysis (Zhang et al., 2012). The phylogenetic tree was constructed by the neighbor-joining method using the MEGA software (version 6.0) with Kimura’s two-parameter calculation model. The topology of the phylogenetic tree was assessed by bootstrap analysis of 1,000 replications.

### Inoculum preparation

The isolates were stored in 15% glycerol at −80°C. Before each experiment, the isolates were thawed and grown individually in 250 mL Erlenmeyer flasks containing 100 mL of LB medium. Each strain was harvested in the late-exponential growth phase by centrifuging (5,000 rpm, 5 min) and washed twice with sterile water, and finally resuspended in MSM, the cell density was adjusted to OD_600 nm_ of 2.0.

### Biodegradation of cypermethrin and 3-phenoxybenzoic acid by pure isolates

To investigate the abilities to degrade cypermethrin or 3-PBA by the isolates, cells were transferred (2% inoculation amount) into 250-mL Erlenmeyer flasks containing 100 mL MSM supplemented with 0.24 mM cypermethrin (and 1 g/L glucose) or 3-PBA. The Erlenmeyer flasks were incubated at 180 rpm and 30°C on a rotary shaker. At certain intervals, an Erlenmeyer flask was removed from the shaker and the cultures were used for growth and chemical analysis. The growth of the strain was determined by measuring the optical density (OD) at 600 nm, the residual cypermethrin concentration was analyzed by UV/VIS (qualitative detection) or GC (quantitative detection), and the 3-PBA concentration was analyzed by UV/VIS or HPLC as described below. All experiments were conducted in triplicates and the results were averages from three independent experiments.

Effect of pH on the degradation of cypermethrin or 3-PBA was tested in MSM, the pH was adjusted to 4.0, 5.0, 6.0, 7.0, 8.0, 9.0, and 10.0 by citrate/Na_2_HPO_4_ buffer (pH 4.0–5.0), Na_2_HPO_4_/NaH_2_PO_4_ buffer (pH 6.0–8.0) and glycine/NaOH buffer (pH 9.0–10.0). Effect of temperature was evaluated at 16, 25, 30, 37, and 42°C, respectively. Effect of salinity (w/v, NaCl concentration) was measured at NaCl concentration (w/v) of 0, 0.5, 1.0, 1.5, 2.0, 2.5, 3.0, 3.5, and 4.0%, respectively. Effect of cypermethrin or 3-PBA concentrations was evaluated at 0, 10, 20, 50, 100, 150, and 200 mg/L.

### Chemical analysis

For cypermethrin detection, the culture was extracted with an equal amount of dichloromethane, the organic layer was dried and redissolved in *n*-hexane. Qualitative detection was carried out using a UV/VIS spectrophotometer (UV-2450, Shimadzu, Japan) at wavelengths from 200 to 340 nm. Quantitative detection was carried out on GC. The GC conditions were as follows: electron capture detector, HP-5 capillary column (30.0 mm × 0.530 mm × 1.5 μm), injector/interface temperature of 240°C, oven temperature of 260°C, detector temperature of 300°C, and N_2_ carrier gas at 1 mL/min. The column temperature was programmed from 150 to 260°C with a rate of 25°C/min. For 3-PBA detection, the sample was dissolved in methanol and analyzed by HPLC using an UltiMate 3000 titanium system (Thermo Fisher Scientific) equipped with a C_18_ reversed-phase column (4.6 mm × 250 mm × 5 μm) (Thermo Fisher Scientific, MA, United States). The mobile phase consisted of acetonitrile, water, acetic acid (50: 49: 1 [vol/vol/vol]) at a flow rate of 0.8 mL/min and a column temperature of 40°C. A VWD-3100 single-wavelength detector was used to monitor the UV absorption with the detection wavelengths being 240 nm, the injection volume is 20 μL. To detect the catabolic metabolites of cypermethrin or 3-PBA, the sample was dissolved in methanol and analyzed by LC-TOF-MS as described by Liu et al. (2021).

### Genome sequencing and genes analysis

Genomic DNA was extracted from the cell pellets of A-3 using a Bacteria DNA Kit (OMEGA). The genome of A-3 was sequenced using the Illumina NovaSeq 6000 by Biozeron Biotechnology Co., Ltd. (Shanghai, China). Gene prediction and annotation were performed by BLAST analysis in the UniProtKB/Swiss-Prot, non-redundant protein (NR), KEGG, and COG databases of the National Center for Biotechnology Information (NCBI) and the Rapid Annotation Subsystem Technology (RAST).

### Data availability

The accession numbers of the 16S rRNA gene sequences of strain JQ-L and A-3 were OP2048080 and OP2048079, respectively; the accession number of the genome of A-3 was JANHNY000000000; the accession numbers of *pobA* and *pobB* were OP219508 and OP219509, respectively; the accession numbers of *proA1* and *proA2* were OP219511 and OP219509, respectively; the accession numbers of *dmpLMNOPQR* were OP219514–OP219520, respectively; and the accession number of *catA2* was OP219513.

## Results

### Enrichment and screening of cypermethrin-degrading strains

In this study, we used cypermethrin as the sole carbon source to enrich cypermethrin-degrading bacteria. It took 7 days to degrade approximately 70% of the added 0.24 mM of cypermethrin for the first round of enrichment. With the progress of acclimation, the ability of the bacterial consortium to degrade cypermethrin was more and more strong. After ten rounds of transfer, the enriched consortium acquired highly efficient cypermethrin-degrading ability. As shown in Supplementary Figure 1, cypermethrin has two characteristic absorption peaks (240 nm and 280 nm) in the UV region (200 to 340 nm). After incubation for 12 h, the two peaks almost completely disappeared, and no new peak was generated. The results indicated that the added cypermethrin was completely degraded by the consortium, and no aromatic metabolite was accumulated.

From the enriched consortium, approximately 20 colonies with different morphologies were isolated and purified. Then, their abilities to degrade cypermethrin were qualitatively determined using UV scanning. The result showed that only one strain, designated as JQ-L, was able to change the UV scanning spectrum of the culture. As showed in Supplementary Figure 2, after treated by strain JQ-L for 60 h, the peak at 280 nm decreased, and a new peak at 300 nm was generated. However, the generated peak did not decrease with prolonged incubation. The result indicated that strain JQ-L transformed cypermethrin to a metabolite which could not be further degraded by strain JQ-L.

Since the enriched consortium could completely degrade cypermethrin, we speculate that there must be strains that could degrade the metabolite produced in the conversion of cypermethrin by JQ-L in the consortium. Therefore, strain JQ-L was co-cultured with other isolated stains to degrade cypermethrin, respectively. UV scanning result indicated that when strain JQ-L was co-cultured with strain A-3, the peaks at 240 nm and 280 nm decreased, and no new peak was accumulated (Supplementary Figure 2). The results indicated that strain A-3 could completely degrade the metabolite generated during cypermethrin transformation by stain JQ-L.

### Identification of strains JQ-L and A-3

Colonies of strain JQ-L grown on LB agar for 5 days were yellow, round, convex, dry with irregular margin, approximately 2–4 mm in diameter (Supplementary Figure 3A). Cells of strain JQ-L were aerobic, Gram-reaction-positive, non-sporeforming, non-motile and rod-shaped (0.5–0.6 μm wide and 1.5–2.0 μm long) (Supplementary Figure 3C). Grew at pH 6.0–9.0 (optimum, pH 7.0), at 15–37°C (optimum, 30°C) and with 0–3.0% (w/v) NaCl (optimum, 1% NaCl). Strain JQ-L utilized glucose, mannose and maltose, but not methanol, did not hydrolyze starch, urea and esculin, positive for the indole test, nitrate reduction and gelatin liquefaction, resistant to bacitracin, but sensitive to kanamycin, tetracycline, streptomycin, erythromycin, clindamycin and gentamicin.

**FIGURE 1:**
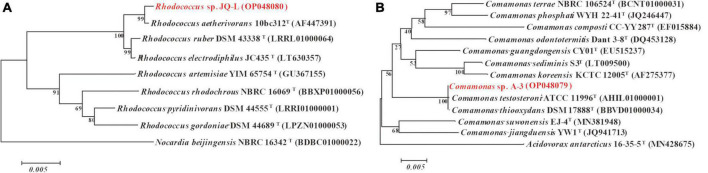
Phylogenetic analysis of strains JQ-L **(A)** and A-3 **(B)** with closely related type strains by neighbor-joining method based on 16S rRNA gene sequences. Bootstrap values (%) are indicated at the nodes, the scale bar represents 0.005 substitutions per site. The GenBank database accession numbers were shown in parentheses.

The almost-complete 16S rRNA gene sequence of strain JQ-L was 1,485 bp. Strain JQ-L showed the highest identity with *Rhodococcus aetherivorans* 10bc321*^T^* (99.9%), and shared 95.5% identities with *R. ruber* DSM43338*^T^* and *R. electrodiphilus* JC435*^T^*. Phylogenetic analysis based on the NJ tree showed that strain JQ-L belonged to the genus *Rhodococcus* and formed a subclade with *R. aetherivorans* 10bc321*^T^, R. ruber* DSM43338*^T^* and *R. electrodiphilus* JC435*^T^* (Figure 1A). According to the phenotype and phylogenetic analysis of 16S rDNA sequence, strain JQ-L was preliminarily identified as *Rhodococcus* sp.

Colonies of strain A-3 grown on LB agar for 5 days were pale yellow, round, convex, moist with smooth edge, approximately 1.5–3 mm in diameter (Supplementary Figure 3B). Cells of strain A-1 were aerobic, Gram-reaction-negative, non-spore forming, and rod-shaped (0.7–0.8 μm wide and 2.0–2.3 μm long), flagellum was observed (Supplementary Figure 3D). Grew at pH 5.0–9.0 (optimum, pH 7.0), at 15–37°C (optimum, 30°C) and with 0–3.0% (w/v) NaCl (optimum, 1% NaCl). Negative for indole test and gelatin liquefaction and positive for nitrate reduction, resistant to kanamycin, streptomycin and penicillin, but sensitive to gentamicin, tetracycline and chloramphenicol. Strain A-3 degraded and utilized 3-PBA, 4-PBA, catechol, phenol and protocatechuate, but not methanol, 2-PBA or diphenyl ether.

The 16S rRNA gene sequence (1,521 bp) of strain A-3 shared 100% identity with *Comamonas thiooxydans* DSM 17888*^T^*. In the phylogenetic tree, strain A-3 was clustered in the genus *Comamonas* and formed a clade with *C. thiooxydans* DSM 17888*^T^* and *C. testosteroni* ATCC 11996*^T^* (Figure 1B). Thus, based on the above analysis, strain A-3 was preliminarily identified as *Comamonas* sp.

### Degradation of cypermethrin by strain JQ-L

**FIGURE 2:**
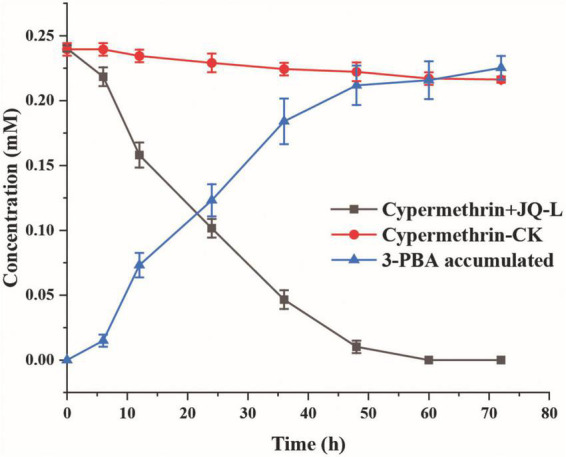
Degradation of cypermethrin by strain JQ-L. CK, 100 mg/L cypermethrin without inoculation; JQ-L, inoculation with strain JQ-L.

The degradation of cypermethrin by strain JQ-L was shown in Figure 2. The results showed that strain JQ-L degraded 79.8% and almost 100% of the added 0.24 mM of cypermethrin within 36 and 60 h of incubations, respectively. Meanwhile, a metabolite with retention time of 4.50 min in HPLC was generated, the retention time of the metabolite was consistent with that of standard 3-PBA (Supplementary Figure 4), MS/MS analysis indicated that the molecular ion peak [M + H]^+^ of this metabolite was 214.0 m/z with fragment ion peaks at 169.1 m/z (Supplementary Figure 5) which was equal to the theoretical molecular mass of 3-PBA. Thus, the metabolite was identified as 3-PBA. During the degradation, the accumulation of 3-PBA continuously increased, its concentration increased to 0.22 mM within 60 h of incubation, and the produced 3-PBA did not decrease within prolonged incubation. These results indicated that strain JQ-L degraded cypermethrin to 3-PBA, and the generated 3-PBA could not be further degraded by strain JQ-L. The optimal conditions for cypermethrin degradation by JQ-L were 30°C, pH 7.0 and 1% NaCl (Supplementary Figure 6). Furthermore, strain JQ-L could also degrade permethrin, fenpropathrin, and deltamethrin, but not bifenthrin (Supplementary Figure 7).

**FIGURE 3:**
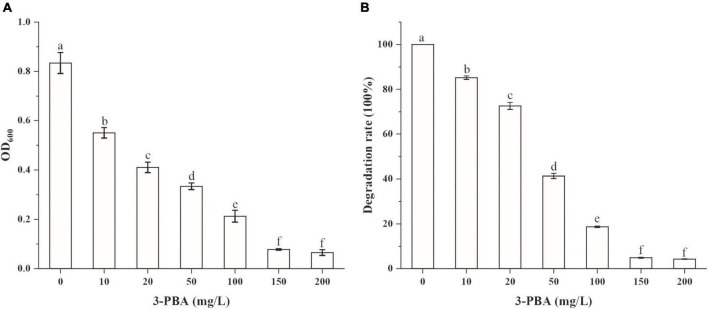
The effect of 3-PBA on the growth of strain JQ-L **(A)** and cypermethrin degradation by strain JQ-L **(B)**. Different lowercase letters indicate significant difference under *P* < 0.05.

To investigate whether the accumulation of 3-PBA affected the cypermethrin-degrading efficiency by strain JQ-L, different concentrations of 3-PBA were added to the culture medium, the growth of strain JQ-L and the degradation of cypermethrin were measured. The results in Figure 3 showed that the addition of 3-PBA significantly inhibited the growth of JQ-L and the degradation efficiency of cypermethrin, and the greater the amount added, the higher the degree of inhibition. When the added 3-PBA reached 100 mg/L, the growth of JQ-L and the cypermethrin degradation were inhibited by approximately 80%. The results indicated that 3-PBA had obvious toxic effect on the growth of JQ-L and cypermethrin degradation.

### Degradation of cypermethrin and 3-phenoxybenzoic acid by strain A-3

**FIGURE 4:**
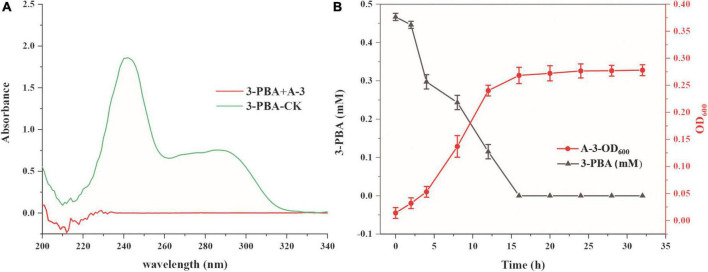
Degradation of 3-PBA by stain A-3. **(A)** UV scanning detection of 3-PBA degradation by strain A-3; **(B)** time course curve of 3-PBA degradation and growth of A-3.

The abilities of strain A-3 to degrade cypermethrin and 3-PBA were investigated. Cypermethrin could not be degraded by stain A-3 (data not shown), and 50–200 mg/L of cypermethrin in the medium had no significant effect on the growth of strain A-3 (Supplementary Figure 8). Figure 4A showed the UV scanning analysis of 3-PBA degradation in MSM by strain A-3. The results showed that the characteristic absorption peaks of 3-PBA at 260 and 300 nm completely disappeared after 48 h of incubation, and no new peak was produced, indicating that strain A-3 completely degraded 3-PBA. Figure 4B showed that strain A-3 completely degraded 0.47 mM of 3-PBA within 15 h of incubation. At the same time, its OD_600 nm_ increased from 0.01 to 0.26, indicating that strain A-3 rapidly degraded 3-PBA and utilized it as the sole carbon source to grow. The optimal conditions for 3-PBA degradation by A-3 were 30°C, pH 7.0 and 1% NaCl (Supplementary Figure 9).

### Degradation of cypermethrin by co-culture of strains JQ-L and A-3

**FIGURE 5:**
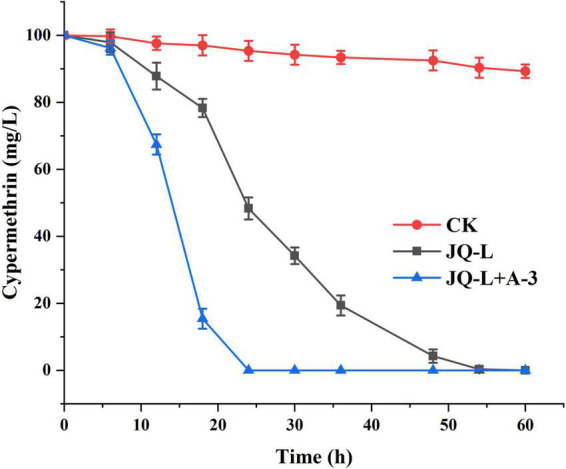
Time course curve of cypermethrin degradation by co-culture of JQ-L and A-3.

The degradation of cypermethrin by the co-culture of strains JQ-L and A-3 was studied. Figure 5 showed that co-culture of JQ-L and A-3 completely degraded 0.24 mM of cypermethrin within 24 h of incubation, while it took approximately 60 h to degraded the same amount of cypermethrin when only JQ-L was inoculated. This result indicated that co-culture of JQ-L and A-3 significantly accelerated the degradation rate of cypermethrin. The possible reason was that A-3 rapidly degraded the generated 3-PBA, thus relieved the toxic effect of 3-PBA on JQ-L.

### Identification of intermediate of 3-phenoxybenzoic acid degradation by *Comamonas* sp. A-3

**FIGURE 6:**
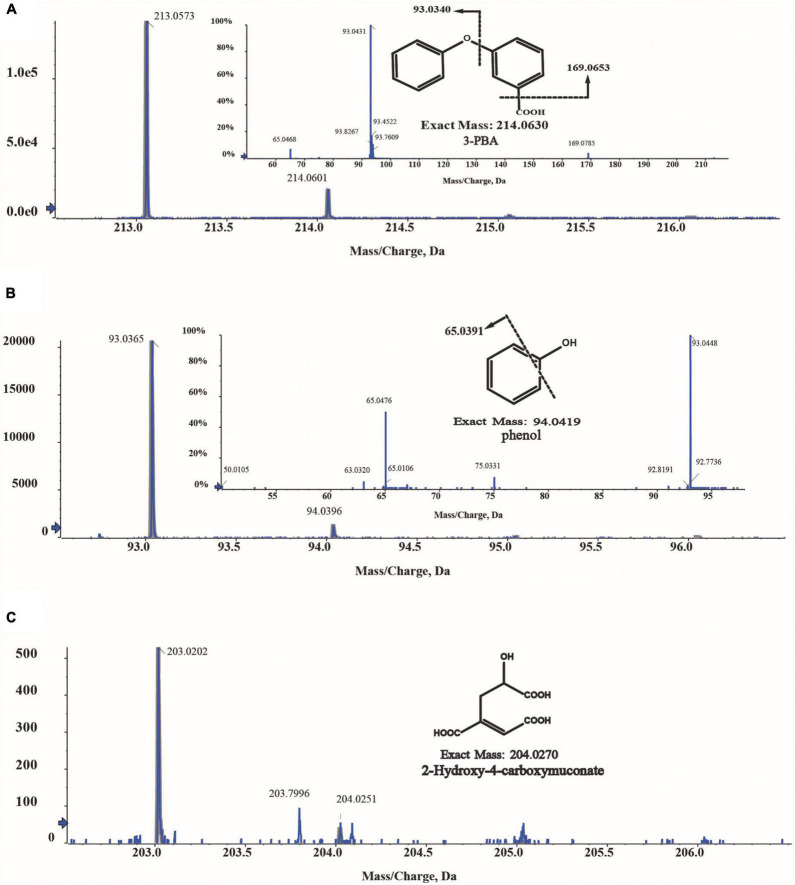
MS/MS analysis of the metabolites of 3-PBA degradation by strain A-3. **(A)** MS/MS analysis of compound I; **(B)** MS/MS analysis of compound II; **(C)** MS/MS analysis of compound III.

The intermediates of 3-PBA degradation by strain A-3 were detected by MS/MS. As shown in Figure 6, three compounds were detected. Compound I showed a molecular-ion peak at m/z 213.05, which was consistent with that of 3-PBA; and its two characteristic fragment ion peaks were also fit to those of 3-PBA (Figure 6A). The molecular-ion peak of compound II (93.03) was fit to that of phenol, and the fragment ion peak (65.03) of compound II was also consistent with that of phenol (Figure 6B). Thus, based on the above analysis, compounds I and II were identified as 3-PBA and phenol, respectively. Compound III showed a molecular-ion peak at m/z 203.02 (Figure 6C), however, its fragment ion peak was not detected.

### Analysis of 3-phenoxybenzoic acid degradation related genes in the genome of A-3

To further investigate the catabolic pathway of 3-PBA in strain A-3, the genome of stain A-3 was sequenced. The genome size of A-3 was 5,511,770 bp, and 5578 putative ORFs were predicted. The genes that possibly involved in catabolism of 3-PBA and other aromatic compounds were predicted by bioinformatics analysis. The results in Table 1 showed that a 3-PBA dioxygenase gene operon *pobAB*, a protocatechuate 4,5-dioxygenase gene operon *proA1A2*, a phenol catabolic gene operon *dmpRKLMNOP*, and a catechol 2,3-dioxygenase gene *catA2* was predicted in the genome of A-3.

**TABLE 1:** Deduced function of each ORF that possibly involved in 3-PBA catabolism in the genome of A-3.

ORF, coding protein	Position, number of amino acid residues	Similar gene (accession number), source	Identity/coverage %
*pobA*, Phenoxybenzoate dioxygenase subunit alpha	Scaffold 27, 409	(Q52185.1), *Pseudomonas oleovorans*	97.3/99
*pobB*, Phenoxybenzoate dioxygenase subunit beta	Scaffold 27, 319	(Q52186.1), *Pseudomonas oleovorans*	99.4/99
*proA1*, Protocatechuate 4,5-dioxygenase alpha chain	Scaffold 11, 149	(P22635.1), *Sphingobium* sp. SYK-6	64.9/78
*proA2*, Protocatechuate 4,5-dioxygenase beta chain	Scaffold 11, 289	(P22636.1), *Sphingobium* sp. SYK-6	60.5/99
*dmpL*, Phenol hydroxylase, P1 oxygenase component DmpL	Scaffold 41, 330	(P19730.1), *Pseudomonas* sp. CF600	46.5/85
*dmpM*, Phenol hydroxylase, P2 regulatory component DmpM	Scaffold 41, 97	(P19731.1), *Pseudomonas* sp. CF600	47.7/87
*dmpN*, Phenol hydroxylase, P3 oxygenase component DmpN	Scaffold 41, 536	(P19732.1), *Pseudomonas* sp. CF600	63.8/94
*dmpO*, Phenol hydroxylase, P4 oxygenase component DmpO	Scaffold 41, 118	(P19733.1), *Pseudomonas* sp. CF600	45.4/99
*dmpP*, Phenol 2-monooxygenase, reductase component	Scaffold 41, 357	(P19734.3), *Pseudomonas* sp. CF600	57.5/99
*dmpK*, phenol hydroxylase component DmpK	Scaffold 41, 63	(P19729.1), *Pseudomonas* sp. CF600	36.4/85
*dmpR*, Positive regulator of phenol hydroxylase, DmpR	Scaffold 41, 563	(Q43965.1), Acinetobacter guillouiae	43.9/95
*catA2*, Catechol 2,3-dioxygenase	Scaffold 41, 314	(Q04285.1), *Pseudomonas putida*	41.3/99

## Discussion

Cypermethrin is a refractory and toxic pesticide that extensively used to control pests in agriculture (Dorman and Beasley, 1991; Katsuda, 1999). At present, a lot of cypermethrin-degrading bacteria have been isolated; however, they usually could only transform cypermethrin to 3-PBA due to lack of downstream catabolic pathway. Furthermore, 3-PBA was toxic and had a feedback inhibition effect, resulting in these bacteria strains often showing relatively low cypermethrin-degrading efficiencies (Chen et al., 2011; Chen et al., 2012a; Liu et al., 2014; Zhao et al., 2016). On the contrary, bacterial consortium has the advantages of species diversity and complete metabolic enzyme system, thus could efficiently and completely mineralized complex refractory compounds (McCoy et al., 2012; Huang et al., 2018). However, the disadvantage of consortium is that it is difficult to culture the consortium in a large-scale and maintain its degradation ability for a long time. Therefore, it is necessary to simplify the consortium to improve the feasibility and reduce the cost. Previous studies have shown that co-culture of cypermethrin-degrading strain and 3-PBA degrading strain could effectively and completely degrade cypermethrin. E.g., compared with strain B-1 alone, the half-life (*t*1/2) of cypermethrin by co-culture of B-1 and 3-PBA-degrading strain SC-1 was shortened from 84.53 to 38.54 h, and approximately 75% of the added 0.24 mM of cypermethrin was degraded by the co-culture within 72 h of incubation (Liu et al., 2014). Chen et al reported that co-culture of *Bacillus cereus* ZH-3 and 3-PBA-degrading strain *Streptomyces aureus* HP-S-01 increased the cypermethrin-degrading efficiency by two times when compared with *Bacillus cereus* ZH-3 alone, the co-culture completely mineralized cypermethrin within 72 h of incubation (Chen et al., 2012b). In this study, a bacterial consortium that capable of efficiently and completely degrading cypermethrin was acclimated using the soil near a pesticide factory. Two strains JQ-L and A-3 were screened from the consortium and identified as *Rhodococcus* sp. and *Comamonas* sp., respectively. The roles of the two strains in the catabolism of cypermethrin were studied. *Rhodococcus* sp. JQ-L transformed cypermethrin to 3-PBA, however, the strain could not further degrade 3-PBA, resulting in the accumulation of 3-PBA in the culture medium, which seriously inhibited the growth and degradation ability of JQ-L. Strain A-3 could not degrade cypermethrin, however, it completely and efficiently degraded and utilized 3-PBA as sole carbon for growth. Co-culture of JQ-L and A-3 could completely degrade cypermethrin. Compared with JQ-L alone, co-culture of JQ-L and A-3 significantly reduced the total degradation time of cypermethrin from 60 to 24 h. Furthermore, in previous reports (Chen et al., 2012b, Liu et al., 2014; Zhan et al., 2020; Zhao et al., 2021), it took 72 h or more for co-culture of cypermethrin-degrading strain and 3-PBA-degrading strain to completely degrade 0.24 mM of cypermethrin, while in this study, it took only 24 h for co-culture of JQ-L and A-3 to completely degrade the same amount of cypermethrin. The possible reasons for the high degradation efficiency of co-culture of JQ-L and A3 were that the two strains were isolated from the same consortium, and their growth conditions were similar, so they could grow well at the some culture system; secondly, JQ-L had enzymes that converted cypermethrin to 3-PBA, while A-3 has enzymes that completely degraded 3-PBA, thus, co-culture of JQ-L and A3 possessed a complete catabolic enzymes system; furthermore, when A3 was co-cultured with JQ-L, it degraded and removed the metabolite 3-PBA thus eliminated the toxicity and inhibition effect of 3-PBA on JQ-L, thereby significantly improving the efficiency of cypermethrin degradation by JQ-L.

**FIGURE 7:**
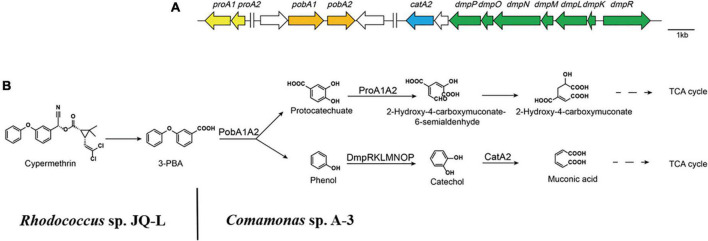
The catabolism of cypermethrin by co-culture of JQ-L and A-3. **(A)** Organization of genes possibly involved in the catabolic pathway of 3-PBA in *Comamonas* sp. A-3. Arrows indicate the size and transcriptional direction of each gene. **(B)** The proposed complete catabolic pathway of cypermethrin by co-culture of *Rhodococcus* sp. JQ-L and *Comamonas* sp. A-3.

3-Phenoxybenzoate is an important intermediate in the synthesis of most pyrethriods and is also the metabolite of their degradation by microorganisms (Huang et al., 2018). 3-PBA belongs to diphenyl ether compound (DE) in structure. DEs are important environmental contaminants, and are highly persistent in environments due to the presence of a diaryl ether linkage. Microbial degradation is one of the most important paths for the dissipation of DEs in the environment (Wang et al., 2014). So far, many 3-PBA-degrading strains were isolated, and the catabolic pathway of 3-PBA have been elucidated (Wang et al., 2014; Zhao et al., 2016; Huang et al., 2018). In all of these reported 3-PBA-degrading strains, the initial degradation step was the cleavage of the diaryl ether. Two 3-PBA cleavage patterns have been identified to date. In *Pseudomonas pseudoalcaligenes* POB310, 3-PBA was cleaved to protocatechuate and phenol under the catalysis of a two-component dioxygenase PobAB (Dehmel et al., 1995); while in *Sphingobium wenxiniae* JZ-1, 3-PBA was cleaved to 3-hydroxybenzoate and catechol under the catalysis of a four-component dioxygenase PbaA1A2BC (Wang et al., 2014). In this study, we found that *Comamonas* sp. A-3 could degrade 3-PBA, catechol, phenol and protocatechuate, and phenol was identified as the metabolite of 3-PBA degradation by A-3. Analysis of A-3 genome indicated that 3-PBA dioxygenase gene *pobAB* but not *pbaA1A2BC* was presented in A-3 genome, indicating that 3-PBA was cleaved to protocatechuate and phenol in A-3 which was similar to that of *P. pseudoalcaligenes* POB310 (Wang et al., 2014). Furthermore, A-3 genome contained two gene operons *proA1A2, dmpRKLMNOP* and a gene *catA2*. Operon *proA1A2* encoded a two-component 4,5-dioxygenase that catalyzed the ring cleavage of protocatechuate (Fuchs et al., 2011), in operon *dmpRKLMNOP, dmpR* encoded a regulatory protein, while *dmpL, dmpM, dmpN*, d*mpO*, and *dmpP* encoded a multiple component phenol monooxygenase catalyzing the hydroxylation of phenol to catechol (Powlowski et al., 1997), and *catA2* encoded a catechol 2,3-dioxygenase, which catalyzed the ring cleavage of catechol to muconic acid (Fuchs et al., 2011). Thus, *proA1A2, dmpRKLMNOP*, and *catA2* together with *pobAB* constituted a complete catabolic pathway of 3-PBA in strain A-3. Based on the substrate spectrum, metabolite identification, and bioinformatics analysis, a complete catabolic pathway of cypermethrin by co-culture of JQ-L and A-3 was proposed in Figure 7, *Rhodococcus* sp. JQ-L transformed cypermethrin to 3-PBA; then in *Comamonas* sp. A-3, 3-PBA was cleaved to protocatechuate and phenol, phenol was hydroxylated to catechol, which was cleaved to muconic acid, and protocatechuate was cleaved to 2-hydroxy-4-carboxymuconate 6-semialdehyde. In the MS/MS analysis of the metabolites of 3-PBA degradation by strain A-3 (Figure 6C), the molecular-ion of compound III was 203.02, which was consistent with that of 2-hydroxy-4-carboxymuconate, the dehydrogenation product of 2-hydroxy-4-carboxymuconate 6-semialdehyde, thus, we inferred that compound III was 2-hydroxy-4-carboxymuconate.

In conclusion, in this study, two bacterial strains *Rhodococcus* sp. JQ-L and *Comamonas* sp. A-3 were screened from a highly efficient 3-PBA-degrading consortium. *Rhodococcus* sp. JQ-L could transform cypermethrin to 3-PBA, which could not be further degraded by JQ-L but was toxic to JQ-L. *Comamonas* sp. A3 could not degrade cypermethrin, but completely degrade 3-PBA. Co-culture of JQ-L and A3 could efficiently and completely degrade cypermethrin and the toxic metabolite 3-PBA. Thus, the two strains have good application potential in the bioremediation of cypermethrin residue-contaminated environments and agriculture products.

## Data availability statement

The datasets presented in this study can be found in online repositories. The names of the repository/repositories and accession number(s) can be found in the article/Supplementary material.

## Author contributions

JH, KZ, and CY conceived the presented idea, contributed to the writing, and prepared the figures and tables. LW, YD, and YY participated in revising the manuscript. All authors contributed to the article and approved the submitted version.
