# Supplementary material for: Highly efficient degradation of cypermethrin by a co-culture of Rhodococcus sp. JQ-L and Comamonas sp. A-3
Source: Front Microbiol. 2022 Sep 16;13:1003820. doi: 10.3389/fmicb.2022.1003820 (PMC9522905; doi:10.3389/fmicb.2022.1003820)
Supplement: Supplementary file 1 [file Data_Sheet_1.docx]

**Running title: Degradation of Cypermethrin by A Co-culture**

**Highly Efficient degradation of Cypermethrin by A Co-culture of *Rhodococcus* sp. JQ-L and *Comamonas* sp. A-3**

Jian He^1, 2^**^**^**, Kaiyun Zhang^2^**^**^**, Lin Wang^1^, Yingchun Du^1^, Ying Yang^1^, Cansheng Yuan^1*^

^1^ College of Rural Revitalization, Jiangsu Open University, Nanjing, China

^2^ Key Laboratory of Agricultural Environmental Microbiology, Ministry of Agriculture, College of Life Sciences, Nanjing Agricultural University, Nanjing, China

^*^Corresponding author

1. mail address: [yuancs@jsou.edu.cn](mailto:yuancs@jsou.edu.cn) (Cansheng Yuan)

**Both authors contributed equally to this work

Tel: +86-25-84396314; Fax: +86-25-84396314


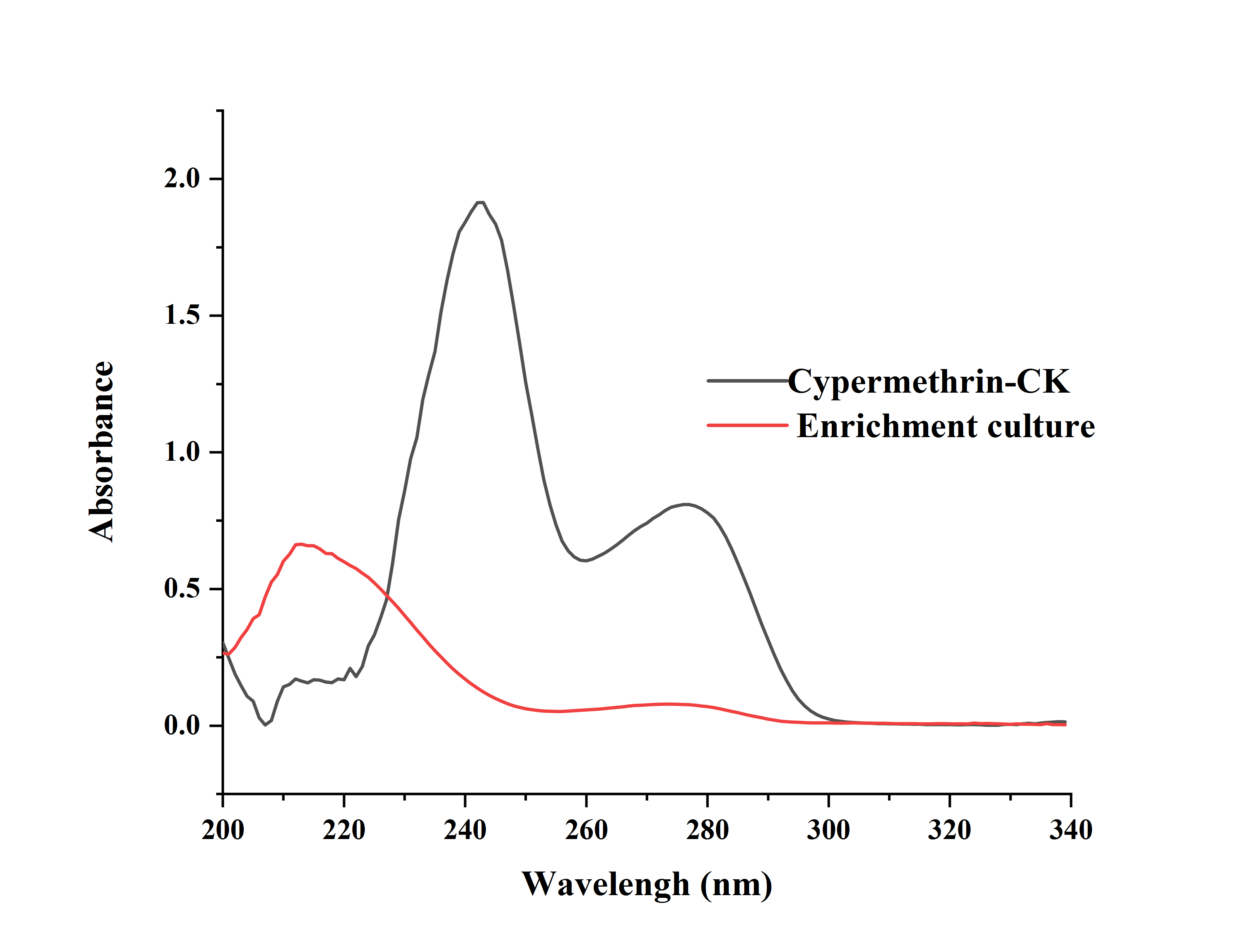


**FIGURE S1 UV scanning detection of cypermethrin degradation by the bacterial consortium.**


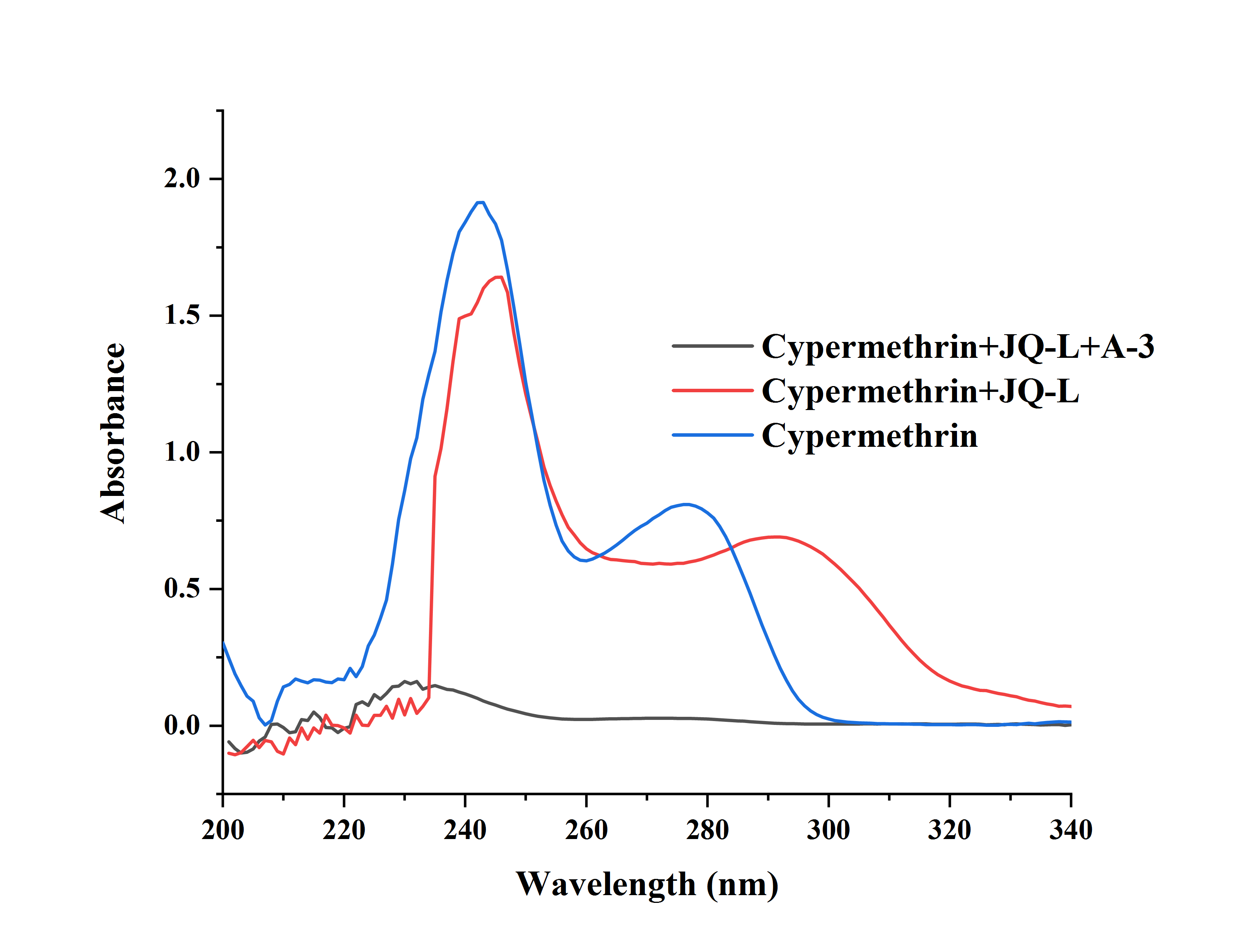


**FIGURE S2 UV scanning detection of cypermethrin degradation by JQ-L and A-3.**


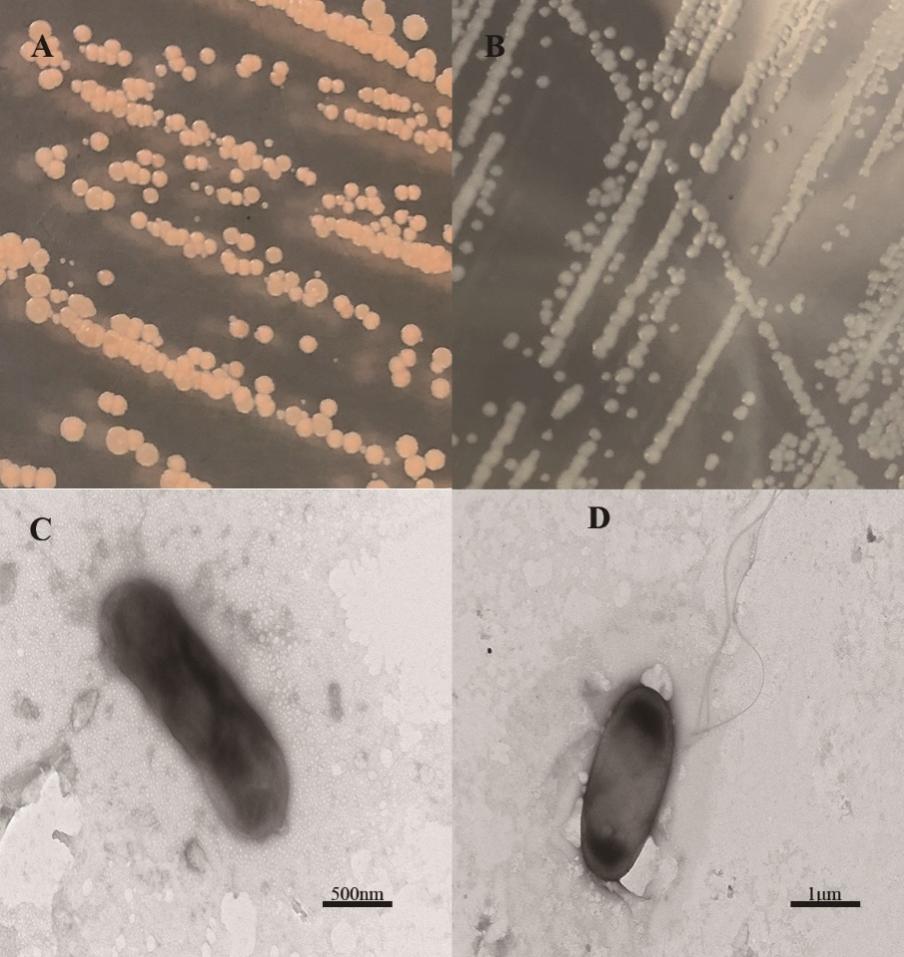


**FIGURE S3 Colony morphologies of strain JQ-L (A) and A-3 (B) on LB plate and electron micrographs of JQ-L (C) and A-3(D)**


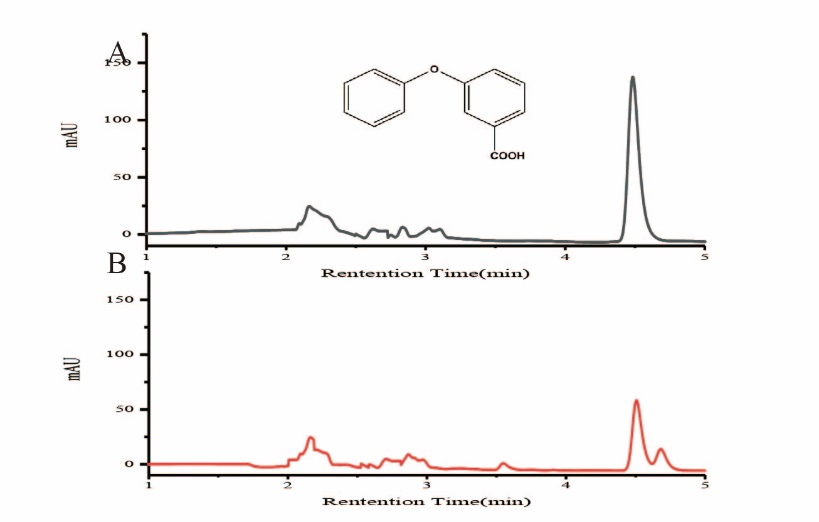


**FIGURE S4 HPLC analysis of the metabolite generated during cypermethrin degradation by JQ-L**

A: 3-PBA standard, B: The metabolite of cypermethrin degradation by strain JQ-L


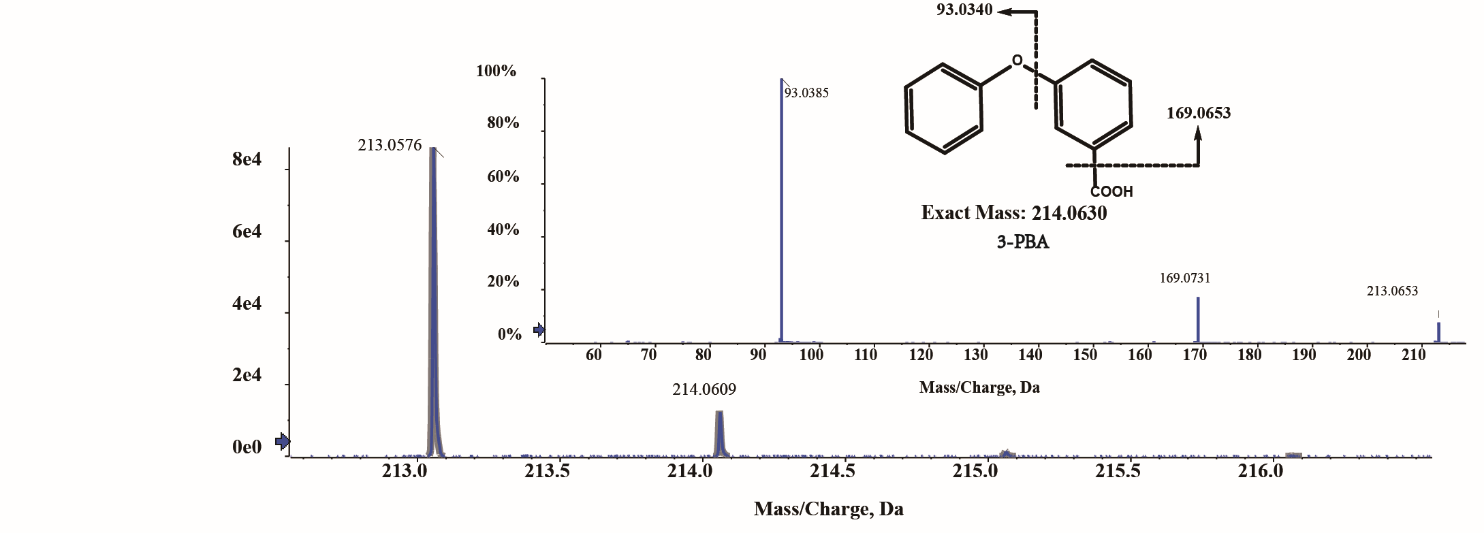


**FIGURE S5 MS/MS analysis of the metabolite generated during cypermethrin degradation by JQ-L**


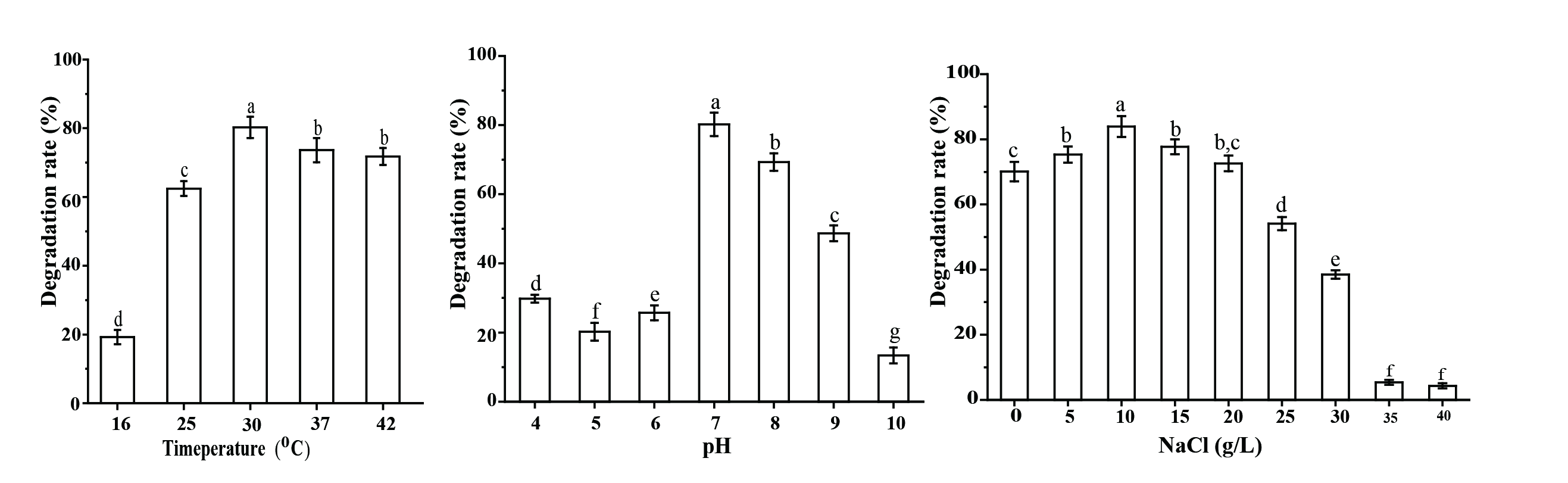


**FIGURE S6 The effect of environmental conditions on the degradation of cypermethrin by strain JQ-L**


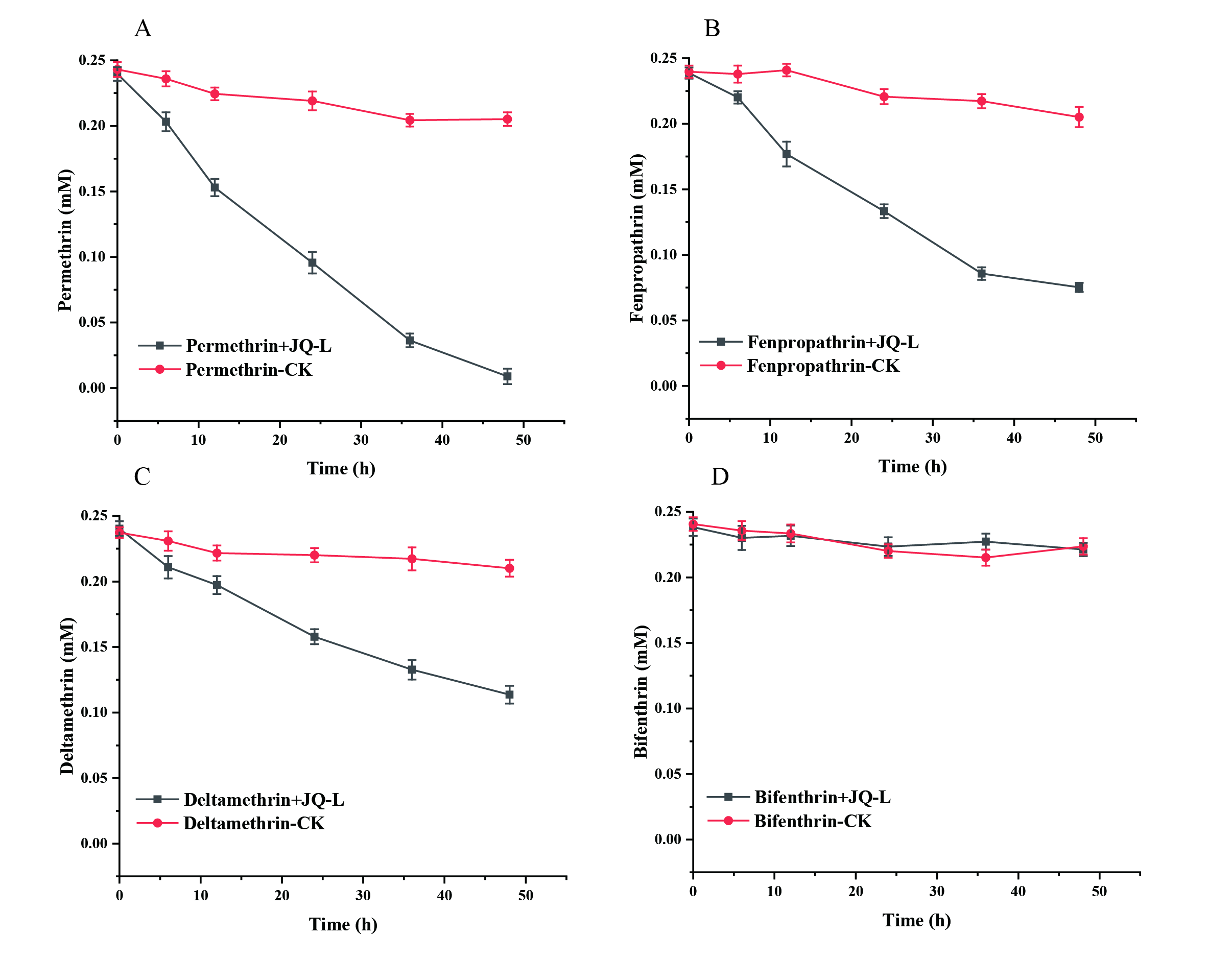


**FIGURE S7 Degradation of different pyrethroid pesticides by strain JQ-L**. A, permethrin; B, fenpropathrin; C, deltamethrin; D, bifenthrin; CK, 0.24 mM of each pyrethroid pesticide without inoculation; JQ-L, inoculation with strain JQ-L.


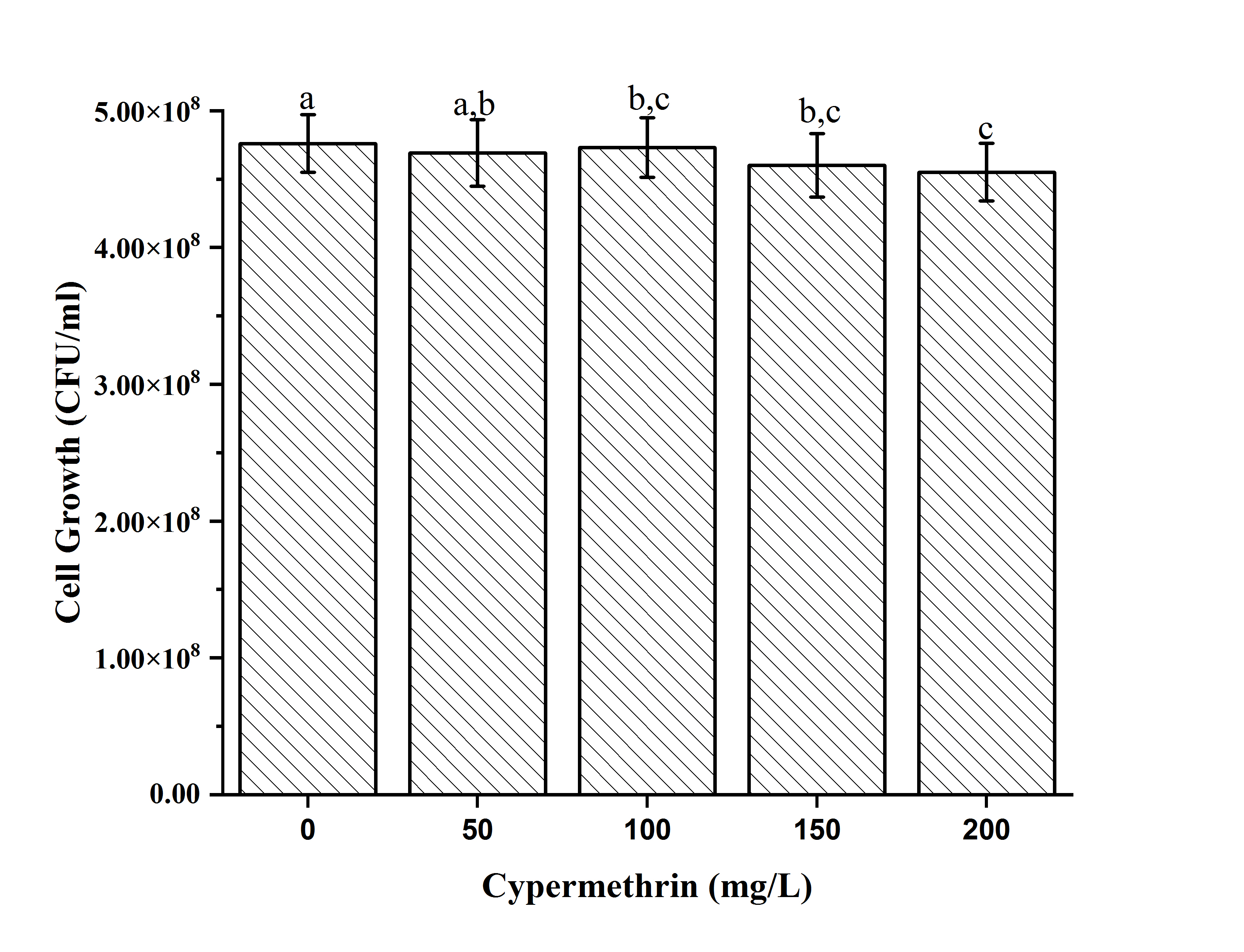


**FIGURE S8 The effect of difficient concentrations of cypermethrin on the growth of strain A-3**


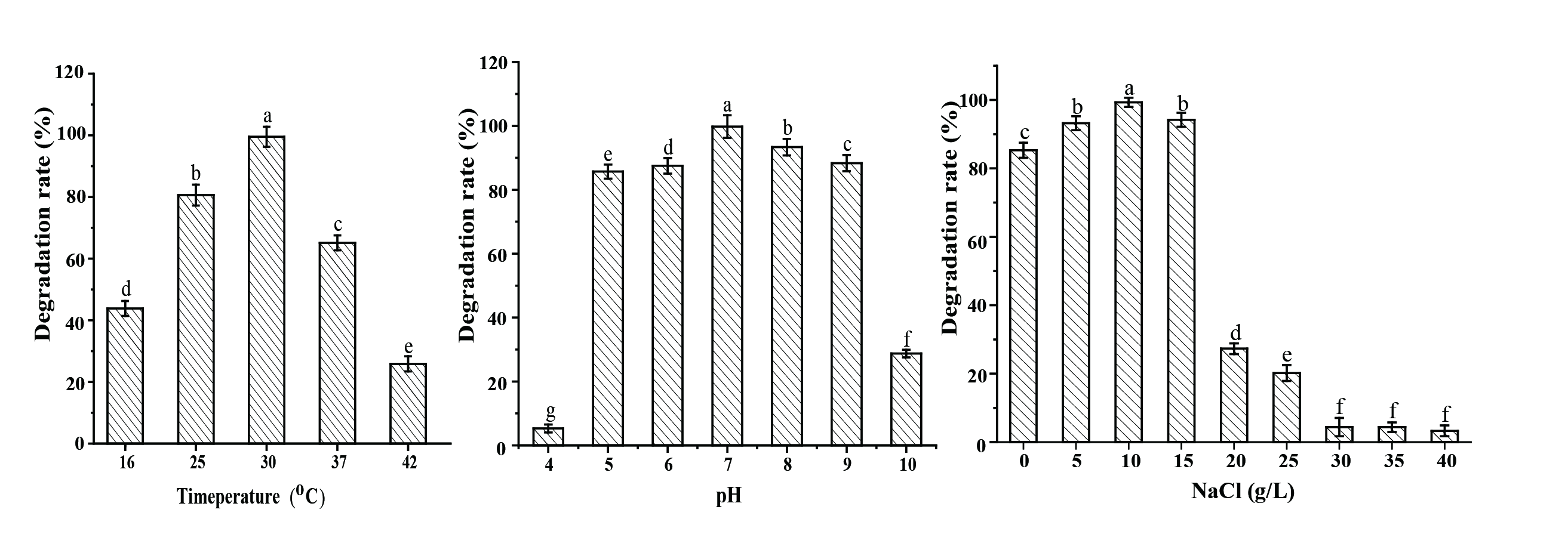


**FIGURE S9 The effect of envieonmental condictions on the degradation of 3-PBA by strain A-3**
